# Supplementary material for: Telomerase mRNA Reduces Radiation-induced DNA Damage of human skin
Source: bioRxiv. 2025 Feb 3:2025.02.01.636031. Preprint. [Version 1] doi: 10.1101/2025.02.01.636031 (PMC11838481; doi:10.1101/2025.02.01.636031)
Supplement: 1 — Supplementary Figure 1. X-ray irradiation induces apoptosis in keratinocytes. (A) Gating strategy for flow cytometric analysis of cell apoptosis in keratinocytes. (B) Flow cytometry analysis reveals the percentage of apoptotic cells among dead keratinocytes 24 hours after exposure to 5 Gy irradiation. (C) Quantitative analysis of the percentage of apoptotic cells among dead keratinocytes, with or without 5 Gy irradiation, 24 hours post-treatment. Supplementary Figure 2. TERT overexpression in HAECs reduces DNA damage and protects cells from bleomycin-induced senescence. (A-B) Representative western blots and densitometric quantification of γH2AX protein levels post-radiation. (C) Quantification analysis of Propidium Iodide (PI)-positive cells, indicative of cell death, following radiation exposure. (D) Representative immunofluorescence staining showing a dose-dependent increase in γH2AX and 53BP1 foci in HAECs and Telo-HAECs treated with bleomycin. (E, F) Representative images and quantitative analysis of SA-β-gal-positive cells in HAECs treated with varying doses of bleomycin. (G) qPCR analysis of TERT expression in HAEC cells treated with different doses of bleomycin. (H-J) Volcano plots illustrate differentially expressed genes in (H) HAEC vs HAEC IR, (I) Telo-HAEC and Telo-HAEC IR, and (J) HEAC vs Telo HEAC. Supplementary Figure 3. TERT overexpression in HAEC reduces DNA damage and increases cell survival. (A) Venn plot depicte pathways associated with differential expressed genes among HAEC-IR vs Telo-HAEC-IR and HAEC vs Telo-HAEC. Heatmap show gene differential expressed genes and common genes from Venn plot in HAEC-IR, Telo-HAEC-IR, HAEC, and Telo-HAEC groups. (B) Gene Set Enrichment Analysis (GSEA) highlights pathway enrichment for DNA damage response and DNA repair in comparisons between HAEC and HAEC IR, as well as Telo HAEC and Telo-HAEC IR. (C) Telomere length analysis by TRF (Terminal Restriction Fragment) assay in HAEC and Telo-HAEC 2h post-radiation. [file NIHPP2025.02.01.636031V1-supplement-1.pdf]

bioRxiv preprint doi: https://doi.org/10.1101/2025.02.01.636031; this version posted February 3, 2025. The copyright holder for this preprint (which was not certified by peer review) is the author/funder, who has granted bioRxiv a license to display the preprint in perpetuity. It is made available under aCC-BY 4.0 International license.

	LA-PCR for Genomic DNA			SA-PCR for Genomic DNA		
**Conditions**	Step 1:	94°C for 2 min	1 cycle	Step 1:	94°C for 2 min	1 cycle
	Step 2:	94°C for 30 sec<br>60°C for 30 sec<br>65°C for 10 min	25 cycle	Step 2:	94°C for 30 sec<br>53°C for 30 sec<br>65°C for 30 sec	20 cycle
	Step 3:	65°C for 10 min	1 cycle	Step 3:	65°C for 10 min	1 cycle
	Hold	4°C	∞	Hold	4°C	∞
	**LA-PCR for Mitochondrial DNA**			**SA-PCR for Mitochondrial DNA**		
**Conditions**	Step 1:	94°C for 2 min	1 cycle	Step 1:	94°C for 2 min	1 cycle
	Step 2:	94°C for 30 sec<br>60°C for 30 sec<br>65°C for 10 min	20 cycle	Step 2:	94°C for 30 sec<br>53°C for 30 sec<br>65°C for 30 sec	20 cycle
	Step 3:	65°C for 10 min	1 cycle	Step 3:	65°C for 10 min	1 cycle
	Hold	4°C	∞	Hold	4°C	∞

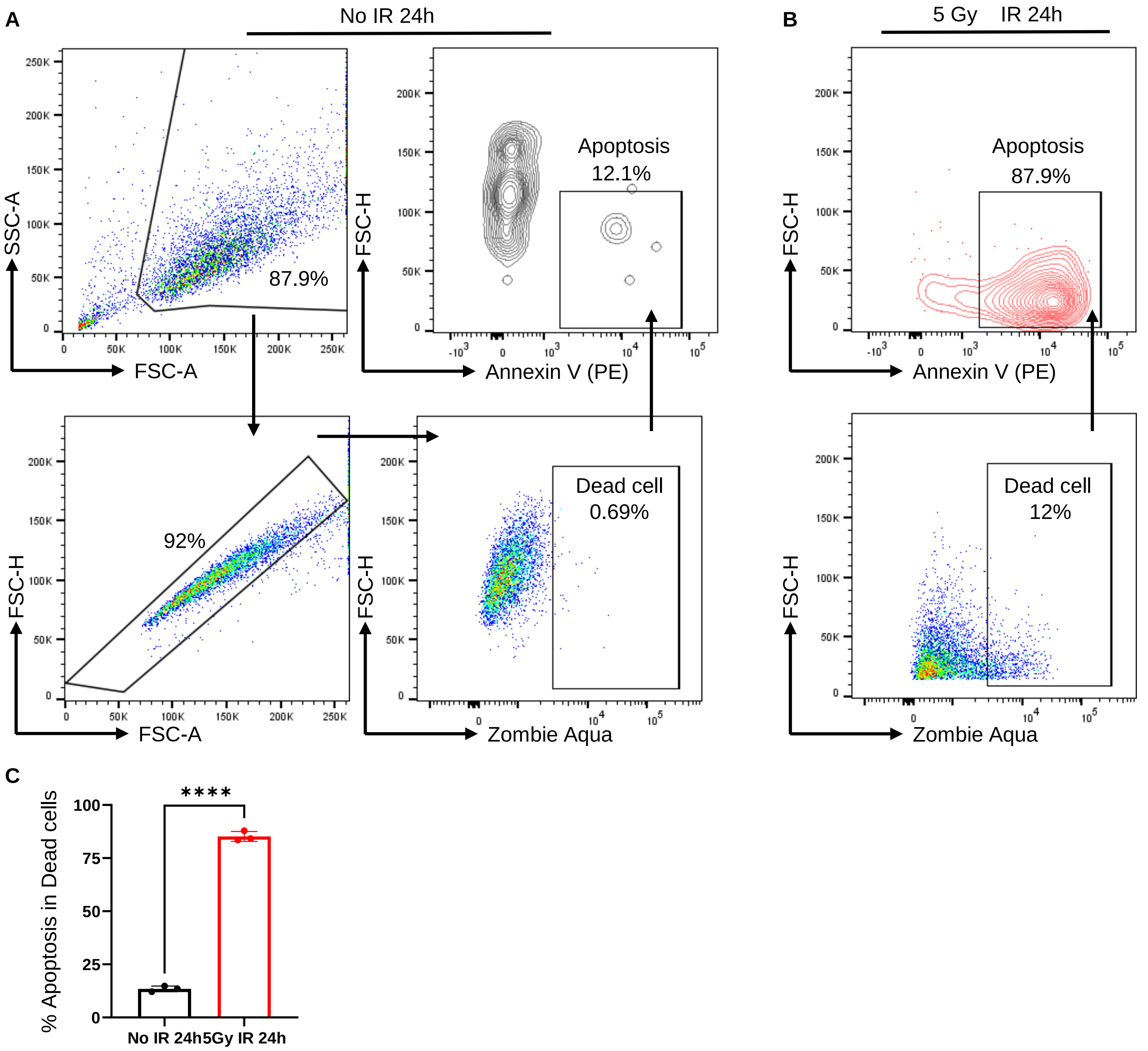

Supplementary Figure 1. X-ray irradiation induces apoptosis in keratinocytes. (A) Gating strategy for flow cytometric analysis of cell apoptosis in keratinocytes. (**B**) Flow cytometry analysis reveals the percentage of apoptotic cells among dead keratinocytes 24 hours after exposure to 5 Gy irradiation. (**C**) Quantitative analysis of the percentage of apoptotic cells among dead keratinocytes, with or without 5 Gy irradiation, 24 hours post-treatment.

bioRxiv preprint doi: https://doi.org/10.1101/2025.02.01.636931; this version posted February 3, 2025. The copyright holder for this preprint (which was not certified by peer review) is the author/funder, who has granted bioRxiv a license to display the preprint in perpetuity. It is made available under aCC-BY 4.0 International license.

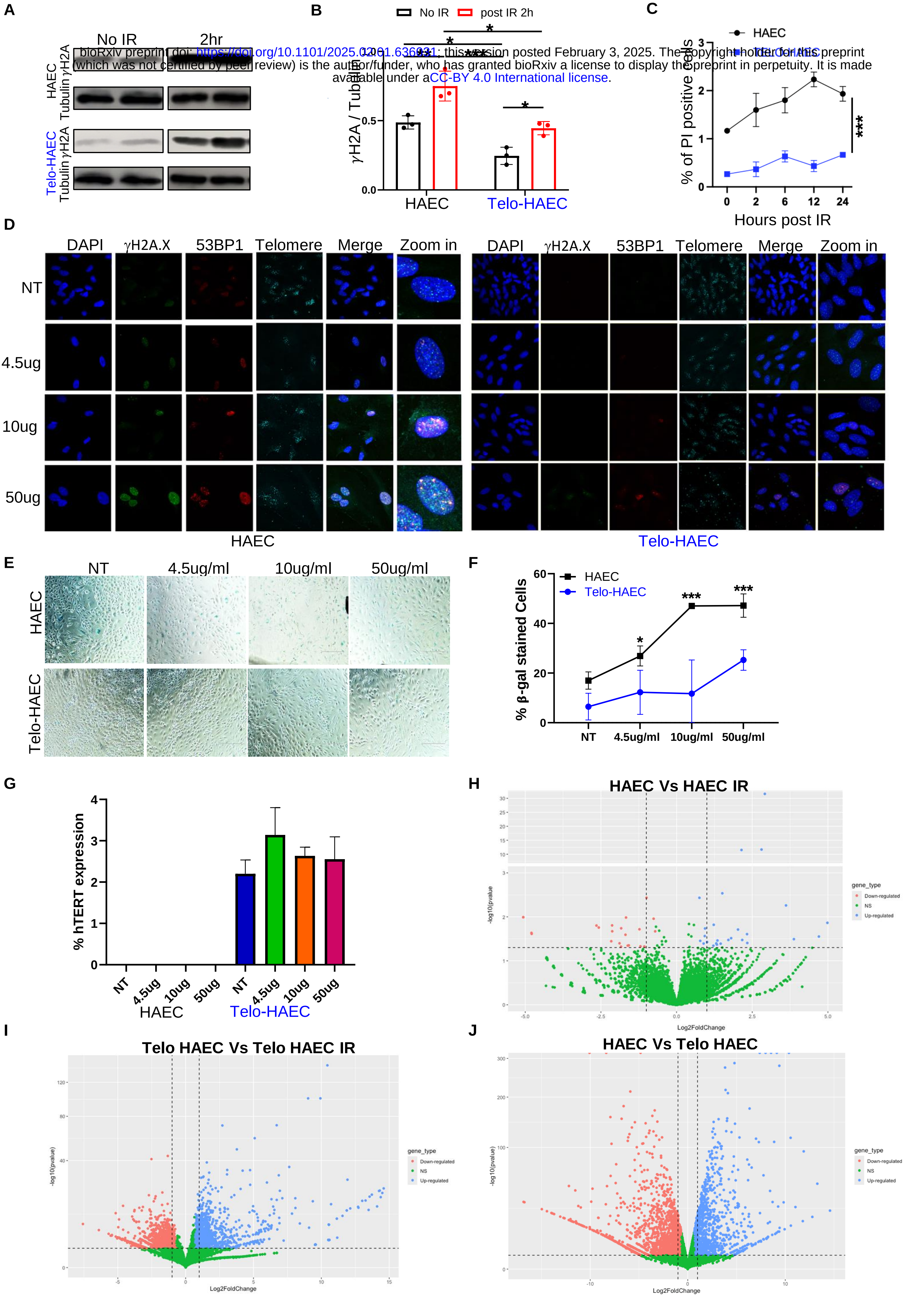

bioRxiv preprint doi: https://doi.org/10.1101/2025.02.01.636031; this version posted February 3, 2025. The copyright holder for this preprint (which was not certified by peer review) is the author/funder, who has granted bioRxiv a license to display the preprint in perpetuity. It is made available under aCC-BY 4.0 International license.

Supplementary Figure 2. TERT overexpression in HAECs reduces DNA damage and protects cells from bleomycin-induced senescence. (**A-B**) Representative western blots and densitometric quantification of γH2AX protein levels post-radiation. (**C**) Quantification analysis of Propidium Iodide (PI)-positive cells, indicative of cell death, following radiation exposure. (**D**) Representative immunofluorescence staining showing a dose-dependent increase in γH2AX and 53BP1 foci in HAECs and Telo-HAECs treated with bleomycin. (**E, F**) Representative images and quantitative analysis of SA-β-gal-positive cells in HAECs treated with varying doses of bleomycin. (**G**) qPCR analysis of TERT expression in HAEC cells treated with different doses of bleomycin. (**H-J**) Volcano plots illustrate differentially expressed genes in (**H**) HAEC vs HAEC IR, (**I**) Telo-HAEC and Telo-HAEC IR, and (**J**) HEAC vs Telo HEAC.

bioRxiv preprint doi: https://doi.org/10.1101/2025.02.01.636031; this version posted February 3, 2025. The copyright holder for this preprint (which was not certified by peer review) is the author/funder, who has granted bioRxiv a license to display the preprint in perpetuity. It is made available under aCC-BY 4.0 International license.

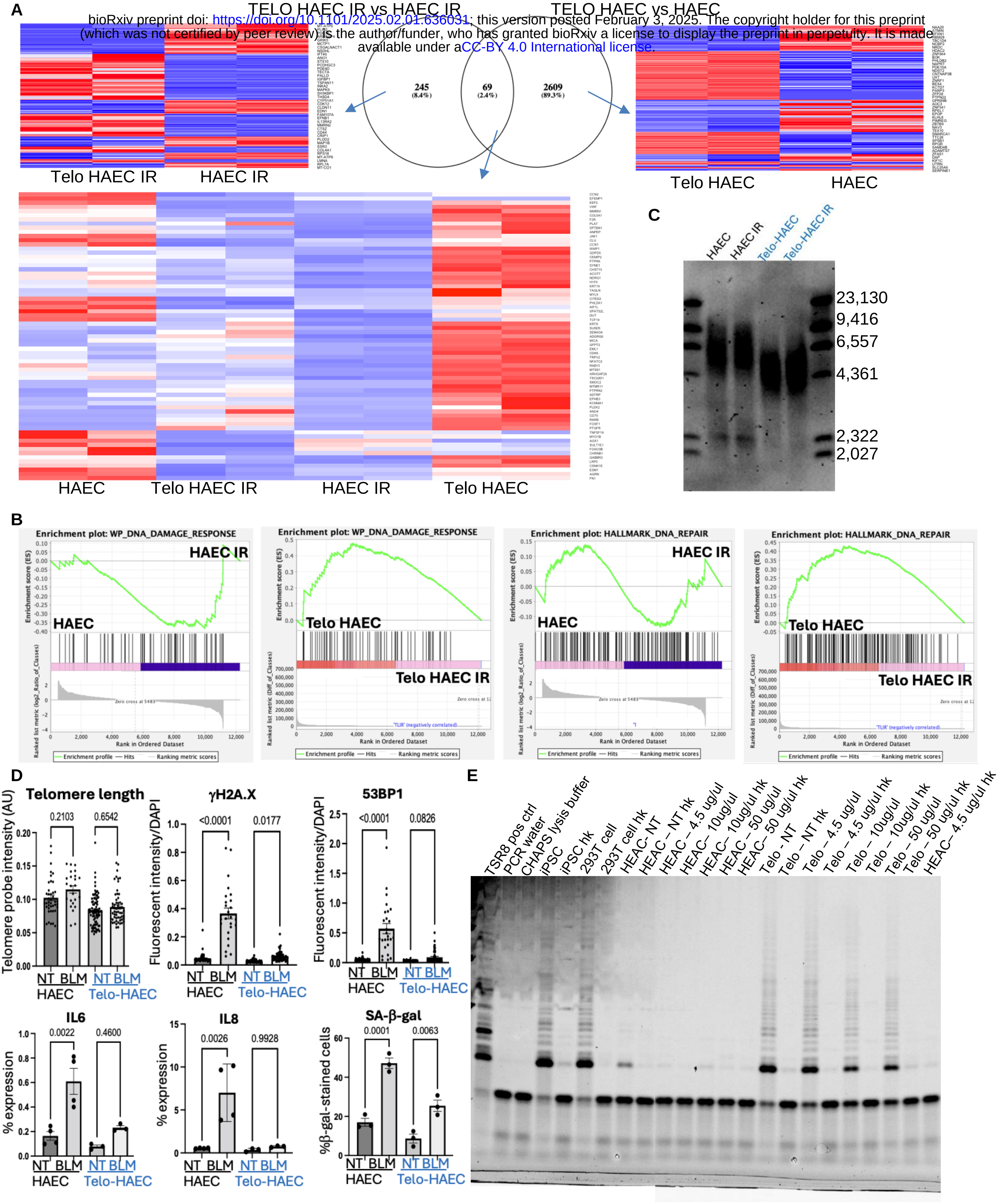

bioRxiv preprint doi: https://doi.org/10.1101/2025.02.01.636031; this version posted February 3, 2025. The copyright holder for this preprint (which was not certified by peer review) is the author/funder, who has granted bioRxiv a license to display the preprint in perpetuity. It is made available under aCC-BY 4.0 International license.

Supplementary Figure 3. TERT overexpression in HAEC reduces DNA damage and increases cell survival. (**A**) Venn plot depicts pathways associated with differential expressed genes among HAEC-IR vs Telo-HAEC-IR and HAEC vs Telo-HAEC. Heatmap show gene differential expressed genes and common genes from Venn plot in HAEC-IR, Telo-HAEC-IR, HAEC, and Telo-HAEC groups. (**B**) Gene Set Enrichment Analysis (GSEA) highlights pathway enrichment for DNA damage response and DNA repair in comparisons between HAEC and HAEC IR, as well as Telo HAEC and Telo-HAEC IR. **(C)** Telomere length analysis by TRF (Terminal Restriction Fragment) assay in HAEC and Telo-HAEC 2h post-radiation. (**D**) qFISH analysis and quantification of γH2A.X, 53BP1, IL-6, IL-8, and SA-β-gal post-bleomycin treatment. (**E**) TRAP assay and qPCR analysis of TERT activity in addition and expression in HAECs and Telo-HAECs following bleomycin treatment.

bioRxiv preprint doi: https://doi.org/10.1101/2025.02.01.636031; this version posted February 3, 2025. The copyright holder for this preprint (which was not certified by peer review) is the author/funder, who has granted bioRxiv a license to display the preprint in perpetuity. It is made available under aCC-BY 4.0 International license.

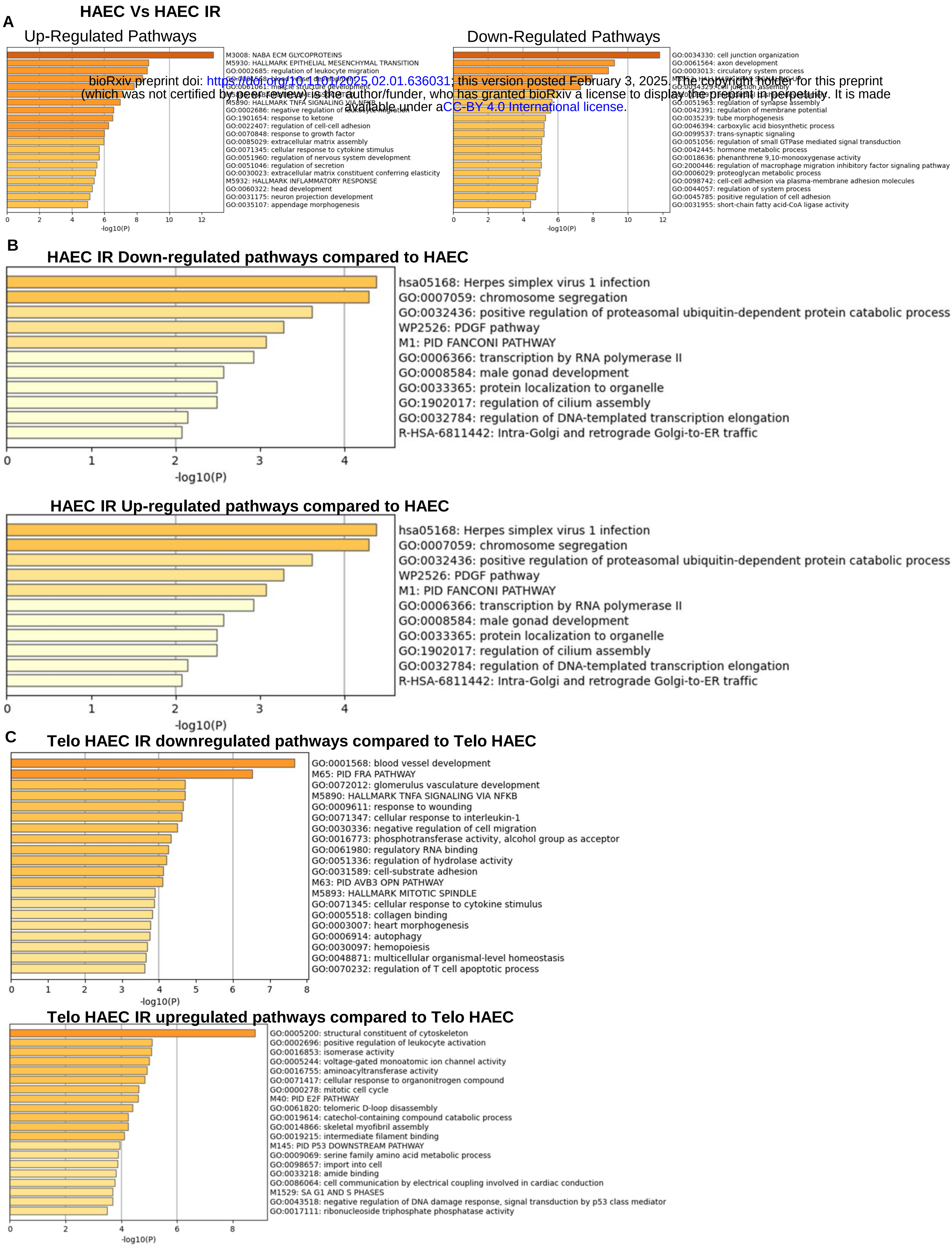

Supplementary Figure 4. Pathway analysis was performed to compare different groups using differentially expressed genes identified from bulk RNA-seq data.

bioRxiv preprint doi: https://doi.org/10.1101/2025.02.01.636031; this version posted February 3, 2025. The copyright holder for this preprint (which was not certified by peer review) is the author/funder, who has granted bioRxiv a license to display the preprint in perpetuity. It is made available under aCC-BY 4.0 International license.

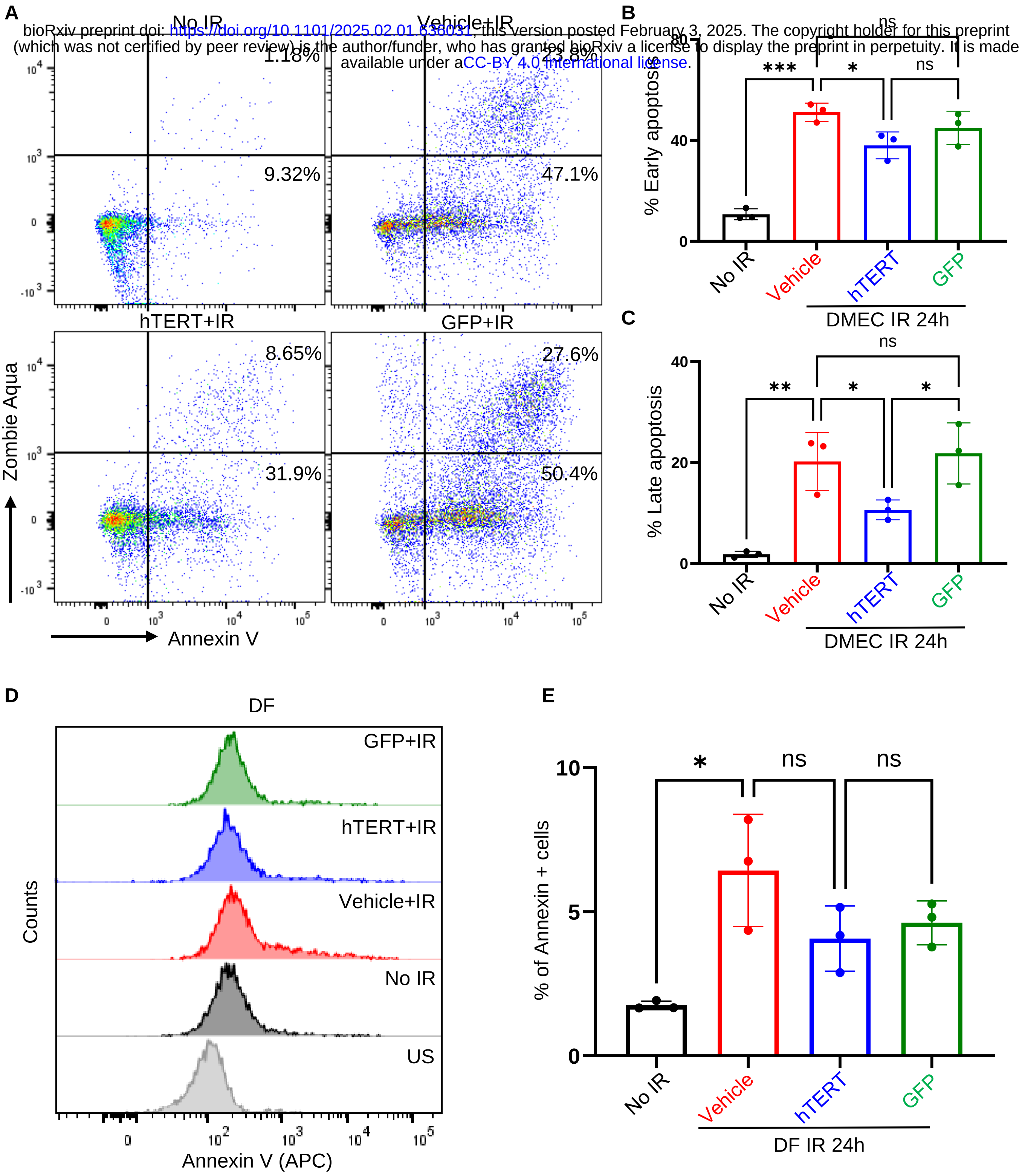

Supplementary Figure 5. TERT mRNA treatment reduces radiation-induced apoptosis in primary skin cells. (**A-C**) Representative flow cytometry plots and quantitative analysis of early and late apoptosis in human dermal microvascular endothelial cells, assessed 24 hours after ionizing radiation (5 Gy) or no irradiation (No IR control). (**D, E**) Representative flow cytometry plots and quantitative analysis of Annexin V-positive cells in indicated groups of human fibroblasts. Data are shown as mean ± SD (n = 3). ns, $P > 0.05$; *, $P < 0.05$; **, $P < 0.01$; ***, $P < 0.001$. *P* values were calculated using two-way ANOVA. DMEC: Dermal Microvascular Endothelial Cells; DF: Dermal Fibroblasts.
